# Effects of assessment method (real-time versus video-recorded) on a validated pain-altered behavior scale used in castrated piglets

**DOI:** 10.1038/s41598-023-45869-8

**Published:** 2023-10-31

**Authors:** Pedro Henrique Esteves Trindade, Magdiel Lopez-Soriano, Victoria Rocha Merenda, Rubia Mitalli Tomacheuski, Monique Danielle Pairis-Garcia

**Affiliations:** 1 Department of Population Health and Pathobiology, College of Veterinary Medicine, North Carolina State University (NCSU), Raleigh, 27607 USA; 2 Anesthesiology Graduation Program, Medical School, São Paulo State University (Unesp), Botucatu, 18618-687 Brazil

**Keywords:** Animal behaviour, Quality of life, Pain

## Abstract

We aimed to compare two assessment methodologies (real-time vs. video-recorded) using the Unesp-Botucatu Pig Composite Acute Pain Scale (UPAPS) in piglets before and after castration. Twenty-nine male piglets were castrated. Four observers scored the UPAPS over three perioperative timepoints of castration following two assessment methodologies. In real-time assessments, the observers were in-person observing the piglets in front of the pen. After two weeks, the observers did video-recorded assessments randomizing piglets and timepoints. Modeling was conducted to compare the UPAPS and each pain-altered behavior between methodologies. Intraclass correlation coefficient (ICC), Bland–Altman, and Lin’s concordance correlation coefficient (CCC) were conducted to investigate agreement between methodologies. UPAPS was statistically equivalent between methodologies (P = 0.4371). The ICC for each method was very good (0.85 to 0.91). The agreement of the UPAPS assessed between methodologies had minimal bias (− 0.04), no proportion bias, and 53% of the assessments presented a perfect agreement. However, CCC of the UPAPS was moderate (0.65), and only one pain-altered behavior (“presents difficulty in overcoming obstacles or other animals”) occurred more in real-time assessments (P = 0.0444). In conclusion, piglet pain assessment by UPAPS can be conducted in real-time based on a suitable agreement between the real-time and video-recorded assessment methods.

## Introduction

From a global perspective, millions of pigs are raised in conditions that can result in individual’s experiencing pain^1,2^. In both commercial farm settings and research laboratories, painful situations can occur as a result of direct pain events inflicted on animals (e.g. tail docking, teeth resection, castration, ear tagging, and notching^2,3^) as well as indirect events resulting in pain experienced as a by-product of disease or management^1,2^.

In addition to the animal welfare implications associated with pain experienced by pigs, *Sus scrofa* is a common model used in translational biomedical research that frequently involves painful procedures such as organ transplantation, stem cell therapy, and endo- and laparoscopic procedures^4^. Failure to effectively identify, assess and treat pain in pigs can significantly influence research outcomes, resulting in potential bias and unpredictable results^5–7^.

Pain assessment in pigs has been studied over the last 30 years in detail^8–17^. Currently, there are five species-specific pain scales used in swine including the Piglet Grimace Scale^18–22^, Sow Grimace Scale^23^, Unesp-Botucatu Pig Composite Acute Pain Scale (UPAPS)^8,24^, and two additional behavioral pain scales^25,26^. In order to accurately assess pain, tools used must demonstrate a high evidence of validation based on robust scientific guidelines such as the Consensus-based Standards for the Selection of Health Measurement Instruments (COSMIN)^27^. According to these guidelines, the UPAPS represents the most robust assessment tool for measuring pain in pigs as demonstrated by its good repeatability and reproducibility, sensitivity, specificity, responsiveness, and excellent internal consistency. In addition, this tool has the discriminatory ability to diagnose pain across ages including newborn^24^ and mature pigs^8^.

Traditionally, the previous pain scales discussed, including the UPAPS, were developed using videos or photos^8,18,22–25^, because it is an essential step needed to conduct masked analyzes and intraobserver reliability^28,29^. However, relying on videos and photos in a research or farm setting is less practical given the logistical considerations such as equipment requirements, labor and time needed to collect, edit and assess videos^7,30,31^. Therefore, to implement pain assessments in a practical way, a real-time assessment approach is needed.

Real-time application of pain scales has been successful in rats^7^ and felines^30^, demonstrating accuracy in the tool and allowing clinicians to provide immediate analgesic intervention for the individuals expressing pain. However, although the UPAPS have been validated for pigs^8,24^, the efficacy of real-time assessment are still unknown.

Therefore, this study aimed to compare two assessment methodologies (real-time vs. video-recorded) using UPAPS in piglets before and after castration. The hypothesis is that there will be no differences between the real-time and video-recorded assessments and both assessments will have suitable agreement.

## Results

**Figure 1:**
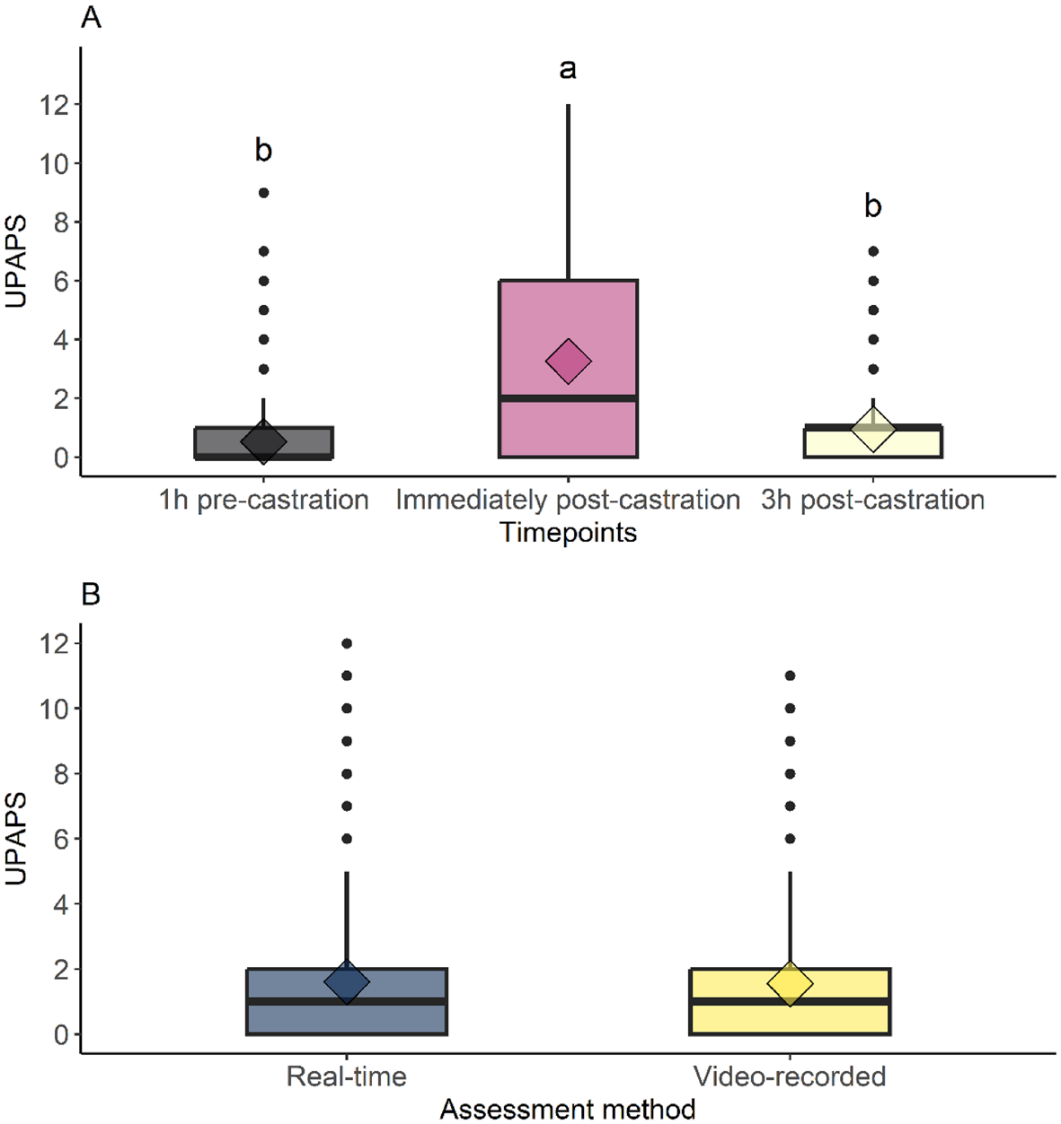
Plots of Unesp-Botucatu Pig Composite Acute Pain Scale (UPAPS) comparing timepoints (**A**) and assessment methods (**B**) (diamond is the mean; different lowercase letters (a > b) indicate statistical difference based on the post-hoc test from multilevel zero-inflated negative binomial model).

The UPAPS presented an overdispersion of zeros based on the histogram (Fig. S1A) and Cameron and Trivedi’s test (Lambda t-test score = 9.55 and P < 0.0001). The count of UPAPS zeros was higher at 1 h pre-castration (n = 154) and 3 h post-castration (n = 110) than immediately post-castration (n = 73), and this unbalance explains the best model considering timepoints in the model logistic component. Assessment methods showed a similar balance of count of UPAPS zeros (n = 140 in real-time and n = 143 in video-recorded) and were not included in the logistic component model (Fig. S1B). Multilevel zero-inflated negative binomial model parameters are depicted in the supplementary material (Table S1). In the post-hoc test, the UPAPS was lower at 1 h pre-castration and 3 h post-castration than immediately post-castration (P < 0.0001) (Fig. 1A) but was statistically equivalent between assessment methods (P = 0.4371) (Fig. 1B). Meanwhile, the UPAPS pain-altered behavior “difficulty overcoming obstacles” occurred more in real-time assessment than video-recorded (P = 0.0444) (Table 1).

**Table 1:** Mean and standard-deviation of Unesp-Botucatu Pig Composite Acute Pain Scale (UPAPS) and its items between assessment methods.

Parameters	Real-time	Video-recorded	P-value
Posture 1	0.07 ± 0.25	0.08 ± 0.27	0.5430
Posture 2	0.02 ± 0.15	0.02 ± 0.15	1.0000
Posture 3	0.09 ± 0.28	0.08 ± 0.27	0.5930
Interaction 1	0.05 ± 0.21	0.07 ± 0.26	0.0880
Interaction 2	0.07 ± 0.25	0.09 ± 0.29	0.1620
Interaction 3	0.05 ± 0.22	0.03 ± 0.18	0.1797
Activity 1	0.09 ± 0.29	0.10 ± 0.30	0.8772
Activity 2	0.01 ± 0.09	0.01 ± 0.12	0.4700
Activity 3	0.07 ± 0.26	0.05 ± 0.22	0.0599
Lift pelvic limb	0.03 ± 0.18	0.04 ± 0.19	0.8158
Scratching rubbing	0.02 ± 0.14	0.02 ± 0.15	0.7677
Walk away/run	0.01 ± 0.11	0.02 ± 0.13	0.4987
Sits with difficulty	0.11 ± 0.32	0.14 ± 0.34	0.2577
Wags tail	0.22 ± 0.41	0.25 ± 0.43	0.2458
Bites grill	0.01 ± 0.08	0.00 ± 0.00	1.0000
Head down	0.15 ± 0.35	0.11 ± 0.31	0.0641
**Difficulty overcoming**	**0.02 ± 0.15**^**a**^	**0.0 ± 0.05**^**b**^	**0.0444**
UPAPS	1.60 ± 2.66	1.55 ± 2.54	0.4390

Multilevel binomial logistic model was used for all behaviors; multilevel zero-inflated negative binomial model was used to the UPAPS; bold is highlighting P < 0.05.

The interobserver reliability of the UPAPS by intraclass correlation coefficient (ICC) was very good in real-time assessment (ICC = 0.92) and in video-recorded assessment (ICC = 0.85) (Table 2).

**Table 2:** Agreement of Unesp-Botucatu Pig Composite Acute Pain Scale (UPAPS) between observers of each assessment method.

Method	Observer	Assessment	ICC		
			Estimate	CI	P-value
Real-time	4	87	0.92	0.89–0.94	2.1^–55^
Video-recorded	4	87	0.85	0.79–0.89	1.61^–32^

ICC is intraclass correlation coefficient; the interpretation of ICC was very good 0.81–1.00; good: 0.61–0.80; moderate: 0.41–0.60; reasonable: 0.21–0.40; or poor < 0.20^32^.

**Figure 2:**
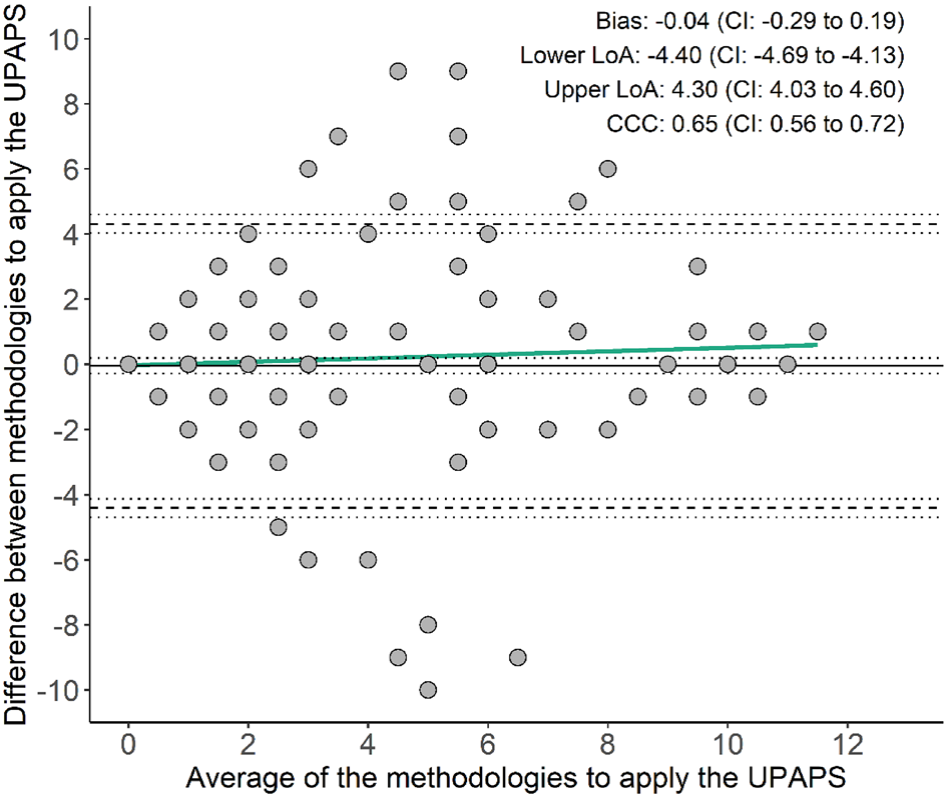
Bland–Altman test of Unesp-Botucatu Pig Composite Acute Pain Scale (UPAPS) assessed in real-time and video-recorded method (LoA is limit of agreement; CI is 95% confidence interval; solid line is the bias; dashed line is the lower and upper LoA; dotted lines is the 95% confidence interval for bias and 90% confidence interval for lower and upper LoA; CCC is Lin’s concordance correlation coefficient; green line is the simple linear model).

The agreement of the UPAPS assessed in real-time and video-recorded methods had minimal bias (− 0.04) and limit of agreement (LoA; − 4.40 to 4.30), and CCC of 0.65 (Fig. 2). The greater majority of the evaluations (53%) showed perfect agreement (no difference) between the two methods, some of the agreements (40%) had differences within the LoA, and only a few evaluations (7%) had differences beyond the LoA. The slope coefficient (β) of the mean between the two assessment methods (β = 0.05; P = 0.2690) was not significant (Table S2), suggesting no proportional bias. The model (χ^2^ = 185.89; P < 0.0001) showed variance not constant (heteroskedasticity behavior) by the Breusch Pagan test and by the pattern in ‘cone’ or ‘V’ format of data distribution in the Bland–Altman plot.

## Discussion

Pain diagnosis is an essential step for pain management and relief in pigs and has the potential to improve the quality of life for millions of pigs experiencing painful procedures in farm and laboratory settings around the world^2,3^. In a translational biomedical research context, the untreated or under-treated pain may act as a confounding factor in the study contributing to poor quality of data and questioning ethical experimental practices^5–7^. As a response to this, objective approaches to assess pain, including the Unesp-Botucatu Pig Composite Acute Pain Scale (UPAPS) are needed to successfully identify and manage pigs experiencing pain^27–29^. However, assessing piglet pain has been traditionally conducted using video recordings which is a less practical approach given the logistical/financial challenges of placing video equipment in such settings and ethical concerns regarding the inability to provide real-time medical intervention and rescue analgesia in suffering animals. Therefore, the objective of this study was to compare two assessment methodologies (real-time vs. video-recorded) on a validated pain-altered behavior scale used in piglets before and after castration.

Behavioral assessment methodology (real-time vs. video-recorded) did not influence total pain scores across timepoints relative to castration. Both methodologies demonstrated sufficient agreement between one another with minimal bias, no evidence of proportional bias, and good interobserver reliability. In addition, most of assessments between methodologies presented perfect agreement, while most differences fell within the estimated limit of agreement (LoA). The results from the current study are in agreement with previous work evaluating real-time methodology assessment for pain evaluation grimace scales in felines^30^ and rats^7^, demonstrating sufficient agreement in pain assessment when utilizing real-time or video-recorded assessment. From a swine-specific perspective, the present study is beneficial as it can permit trained laboratory technicians and swine farm caretakers to assess pain in swine in real-time thus providing a means to assess, diagnose and treat pain conditions in a timely manner.

Although total pain score assessment was not different, it should be noted that the Lin’s concordance correlation coefficient (CCC) between methodologies was only moderate, and the variance of the data was inconsistent (heteroskedasticity). Based on bias, observers in this study slightly underestimated total pain scores using real-time assessment. These results are in agreement with previous work reported in felines^30^ and rats^7^. These deviations also mirror work conducted in mice with grimace scales that demonstrated lower total pain scores using real-time assessments compared to photographs edited from video-recordings^31^. These minor disagreements between assessment methodologies may be partially explained by observer fatigue and attention. Real-time assessment requires constant attention to the piglet in a distracting environment, which may be a potential source of fatigue as reported in previous studies^7,30,31^. In this situation, environmental conditions (e.g., sound, movement, other activities) could have influenced the observer making them less accurate in observing subtle individual behaviors. In contrast, video-recorded assessment was performed in the laboratory or personal office with minimal to no distractors and observers had the option to watch the video repeatedly and take short breaks in between video assessment, thus potentially explaining why real-time assessment underestimated scores compared to recorded. Additional work is needed to understand what additional factors influence this minor bias, including future studies looking at the impact of observer gender and experience on bias.

This study is not free of limitations. The number of times each observer watched each video was not recorded and the number of videos per day or hour was not standardized and these issues may have benefited the video-recorded assessments in comparison with real-time assessments. In addition, the presence of the human may have influenced the expression of pain-altered behaviors as a response to stress-induced analgesia^33,34^, thus influencing total pain scores for both methodologies.

Practical implications of the study include facilitating pain diagnosis by the use of the UPAPS in real-time in laboratory and farm settings, enabling immediate pain diagnosis and medical intervention. Also, the findings presented herein may benefit the approval process for drugs to relieve pain in swine that can only be labeled if they are proven efficacious using validated tools, and currently, in the United States there are no label-approved drugs to relieve pain in swine. Future studies may analyze the effect of experience, gender, training, and cultural aspects of the observers in the pain-altered behaviors in other painful conditions.

In conclusion, piglet pain assessment by UPAPS can be conducted in real-time based on suitable agreement between the real-time and video-recorded assessment methods, however, strategies need to be devised to overcome the smooth deficiencies in agreement between both methods.

## Materials and methods

This study was approved by the Institutional Animal Care and Use Committee of North Carolina State University (IACUC protocol 20-113). Animals were cared for and handled in accordance with the Guide for the Care and Use of Agricultural Animals in Research and Teaching^35^. This study was conducted on a commercial sow farm located in the Southeastern United States as part of a larger study^36^. No animals were castrated exclusively for the purposes of this study, the piglets’ castration was a regular routine of the farm, which contributes with two of the four R’s of animal experimentation (reduce and responsibility^37,38^). Recognizing castration is painful, all enrolled piglets received pain management before the procedure. The study is reported in accordance with ARRIVE guidelines.

### Animals, housing, and procedures

A total of 29 Large White x Duroc cross male piglets from 15 l (1.9 piglets per litter average) were enrolled in this study. Prior to enrollment, piglets were assessed using enrollment criteria described in Table 3. All male piglets were enrolled in each litter for the study (range 1–10 males enrolled per litter). Male piglets meeting all enrollment criteria were then weighed and individually identified using a permanent marker on the back. Castration was performed on each piglet by one trained farm employee. Twenty minutes prior to castration, all piglets received the following pain-control protocol: 3 ml of 2% buffered Lidocaine HCl injectable solution (Lidocaine Hydrochloride, Covetrus, Dublin, Ohio, US) administered intra-inguinal (1.5 ml per inguinal canal) and 2.2 mg/kg of flunixin meglumine (Banamine®, Merck Animal Health, Madison, NJ, US) administered intranasally. Piglets were then placed in dorsal recumbency, and two vertical incisions were made using a scalpel blade. Once the incisions were made, testicles were exposed, spermatic cords cut, and testicles were completely removed by traction.

**Table 3:** Inclusion and exclusion criteria utilized for piglets at the time of enrollment.

Inclusion criteria	Exclusion criteria
Two to five days of age^a^	Clinical signs of disease
Intact tails	Treatment with any type of antibiotic^b^
Both testicles descended	
Body weight greater than 0.5 kg Maintained within litters with at least five additional male siblings	

^a^Cross-fostering was permitted prior to enrollment in the study.

^b^Sows nursing the litter that received any type of antibiotic were excluded from enrollment.

Piglets were housed with sows on fully slatted, tunnel ventilated farrowing rooms. Room temperature was managed through a computerized control system at 22° ± 1.0 °C for the sow and heat mats for piglets were set to approximately 30–35 °C. Within each room, sows and litters were housed in individual farrowing crates (2.5 × 0.7 m) with additional space for piglets (2.5 × 1.3 m) surrounding the crates. Lighting was turned on between 06:00 h and 16:30 h.

### Behavioral pain scale

Pain scores were quantified utilizing the previously validated Unesp-Botucatu Pig Composite Acute Pain Scale (UPAPS) for pre-weaned piglets^8,24^ (Table 4). The pain scale consisted of five behavioral items, with each item sub-categorized into four descriptive levels. A numerical score was designated from ‘0’ to ‘3’, with a ‘0’ representing normal behavior (free of pain) and '1’, ‘2’ and ‘3’ corresponding to proportional to pain intensity pronounced behavioral deviation. The total pain score of UPAPS (0–15) was considered to assess pain in the previous studies^8,24^. Behavior was assessed continuously for 4-min at the following three timepoints: 1 h before castration, immediately post-castration, and 3 h post-castration. The timepoints were chosen based on the pain intensity reported by previous studies assessing pain in swine^8,24,36,39^. Before castration, it is expected that pigs will not be experiencing pain. Moderate to intense pain is expected immediately post-castration, while at 3 h hours post-castration pigs are expected to be pain free or feel mild pain.

**Table 4:** The Unesp-Botucatu Pig Composite Acute Pain Scale (UPAPS) for scoring pain in piglets^8,24^.

Item	Score	Score/criterion	Links to videos
Posture	0	Normal (any position, apparent comfort, relaxed muscles) or sleeping	https://youtu.be/QSosCD2SD4E
	1	Changes posture, with discomfort	https://youtu.be/SpaWsFCrPxE
	2	Changes posture, with discomfort, and protects the affected area	https://youtu.be/VjSlsRrG8yA
	3	Quiet, tense, and back arched	https://youtu.be/pm4hJ5163ao
Interaction and interest in the surroundings	0	Interacts with other animals; interested in the surroundings or sleeping	https://youtu.be/-880STgYq2I
	1	Only interacts if stimulated by other animals; interested in the surroundings	https://youtu.be/nXjOdwn3dyw
	2	Occasionally moves away from the other animals, but accepts approaches; shows little interest in the surroundings	https://youtu.be/2k2JDr5U6As
	3	Moves or runs away from other animals and does not allow approaches; disinterested in the surroundings	https://youtu.be/se70oYXcWFw
Activity	0	Moves normally or sleeping	https://youtu.be/cC75t7L5-YA
	1	Moves with less frequency	https://youtu.be/lQo9wq8LAn8
	2	Moves constantly, restless	https://youtu.be/YQRJjijLvpk
	3	Reluctant to move or does not move	https://youtu.be/Zyx0G3Wpt8o
Attention to the affected area		A. Elevates pelvic limb or alternates the support of the pelvic limb	https://youtu.be/UD99ftO7HE0
		B. Scratches or rubs the painful area	https://youtu.be/7idfFk1harE
		C. Moves and/or runs away and/or jumps after injury of the affected area	https://youtu.be/u-Pqubom278
		D. Sits with difficulty	https://youtu.be/ETNEOCVV4h0
	0	All the above behaviors are absent	
	1	Presence of one of the above behaviors	
	2	Presence of two of the above behaviors	
	3	Presence of three or all the above behaviors	
Miscellaneous behaviors		A. Wags tail continuously and intensely	https://youtu.be/pU5dGZFNRHc
		B. Bites the bars or objects	https://youtu.be/cF3dsq7gMtk
		C. The head is below the line of the spinal column	https://youtu.be/ZcIgngclRpI
		D. Presents difficulty in overcoming obstacles (example: another animal)	https://youtu.be/HlvdOI3lGuY
	0	All the above behaviors are absent	
	1	Presence of one of the above behaviors	
	2	Presence of two of the above behaviors	
	3	Presence of three or all the above behaviors	

### Observers and training

A total of four observers were used to collect pain assessment scores over the course of the trial. All observers were veterinary medicine students, currently enrolled at the university within the second or third year of the program. Two of the observers self-identified as females and two as males, one of each gender had familiarity with the swine industry and the other had no familiarity.

Prior to on-farm data collection, all observers underwent three 2 h training sessions conducted by one of the co-authors with previous experience with UPAPS (MLS). During this session, the trainer provided video examples of each behavior with written definitions and descriptions. Following this training, observers scored 20 4-min videos of piglets in pain (post-castration) and pain-free (pre-castration) using the UPAPS. Videos were assessed in random order and observers were masked to piglet state (pain or pain-free). These videos were different from the ones used for the assessments in the present study. The observers had a very good level of agreement (0.917 [0.839 to 0.964 95% confidence interval]) with the trainer based on the intraclass correlation coefficient.

### Pain assessment method

The behavioral pain scale was assessed using two behavioral methodologies.

#### Real-time assessment

Observers stood within 30 cm of the perimeter of the farrowing crate to observe the piglets. Two observers were at the front and two at the back of the crate. Observers were quiet, had minimum movements and had little to no contact with the piglets. Each piglet was observed for 4 min per timepoint.

#### Video-recorded assessment

Video was recorded in parallel with real-time assessment using a high-definition camera (Sony HDR-CX405®; New York, NY, USA) placed on a tripod approximately 30 cm from the crate at a 122-cm height. The positioning of the camera was the position of the observers during real-time assessment at the front of the crate. A total of 177 videos were obtained (29 per timepoint, 348 min of video-recorded in total; each piglet was filmed for 4 min per timepoint). No video edition was performed. Video clips were assessed for quality and then masked by a senior researcher (MPG) who did not perform any video-recorded assessment. Video order was randomized for each observer and video-recorded assessments occurred 15 days following real-time assessment.

### Statistical analysis

Data were analyzed using R software within the integrated RStudio environment (Version 4.1.0; 2021-06-29; RStudio, Inc., Boston, MA, USA). The functions and packages used were presented in the format ‘package::function’ corresponding to the computer programming language in R. A significance of 5% was considered for all tests. A palette of colors distinguishable by people with common forms of color blindness was used in all figures (ggplot2::scale_colour_viridis_d).

Modeling was conducted to compare real-time pain assessments *versus* video-recorded considering other effects of experimental design. The histogram plot (stats::hist) and Cameron and Trivedi’s test (overdisp::overdisp) proved the overdispersion (excess of zeros) in UPAPS, requiring a zero-inflated model. Zero-inflated models combine logistic and count distributions in the fixed effects of the same model for a better fit of the data^40^. Then, a multilevel zero-inflated negative binomial model (glmmTMB::glmmTMB) was identified as the best fit compared with other models according to the Bayesian information criterion (stats::BIC). The UPAPS was used as the response variable, while the assessment methods, and timepoints were used as explanatory variables in the model count component (negative binomial distribution). Timepoints were included as explanatory variables in the model logistic component (Bernoulli distribution). Piglets nested within each litter and observers were included as random effects of the model. In addition, the all behavioral items from the UPAPS were converted into dummy variables (0 = absence and 1 = presence of each level of each item) (fastDummies::dummy_columns) and used as response variable in a multilevel binomial logistic model (lme4::glmer) with the same fixed effects of the model count component and random effects described previously. For all models, the Bonferroni was used for adjusting the multiple comparisons in the post-hoc test (lsmeans::lsmeans and multcomp::cld).

Intraclass correlation coefficient (ICC), two-way random effects model, type agreement multiple observers/measurements, and its 95% confidence interval (CI) (irr::icc) were used to assess the interobserver reliability of the UPAPS.

Bland–Altman test for repeated measures and Lin’s concordance correlation coefficient (CCC) (SimplyAgree::agree_reps) were used to verify the agreement of UPAPS assessed in real-time and video-recorded method. Bland–Altman analysis is enabled to detect the bias referring to the difference between two measurement methods^41^. In addition, the Bland–Altman analysis provides the limit of agreement (LoA), which indicates the expected range that most differences between methods should occur^41^. A simple linear regression (stats::lm) was conducted to analyze the proportion bias between both assessment methods. Proportional bias represents an increase in the difference between the methods evaluated at higher or lower UPAPS. Then, the difference of UPAPS between the two assessment methods was used as a response variable and the mean of UPAPS between the two methods was used as an explanatory variable. Heteroskedasticity was tested by Breusch Pagan test (olsrr::ols_test_breusch_pagan).

### Supplementary Information

Supplementary Information 1.

Supplementary Information 2.

## Data Availability

All data analysed during this study are included in its Supplementary Information files.
