# Supplementary material for: Effects of assessment method (real-time versus video-recorded) on a validated pain-altered behavior scale used in castrated piglets
Source: Sci Rep. 2023 Oct 31;13:18680. doi: 10.1038/s41598-023-45869-8 (PMC10618161; doi:10.1038/s41598-023-45869-8)
Supplement: Supplementary file 2 — Supplementary Information 2. [file 41598_2023_45869_MOESM2_ESM.docx]

**Supplementary material**


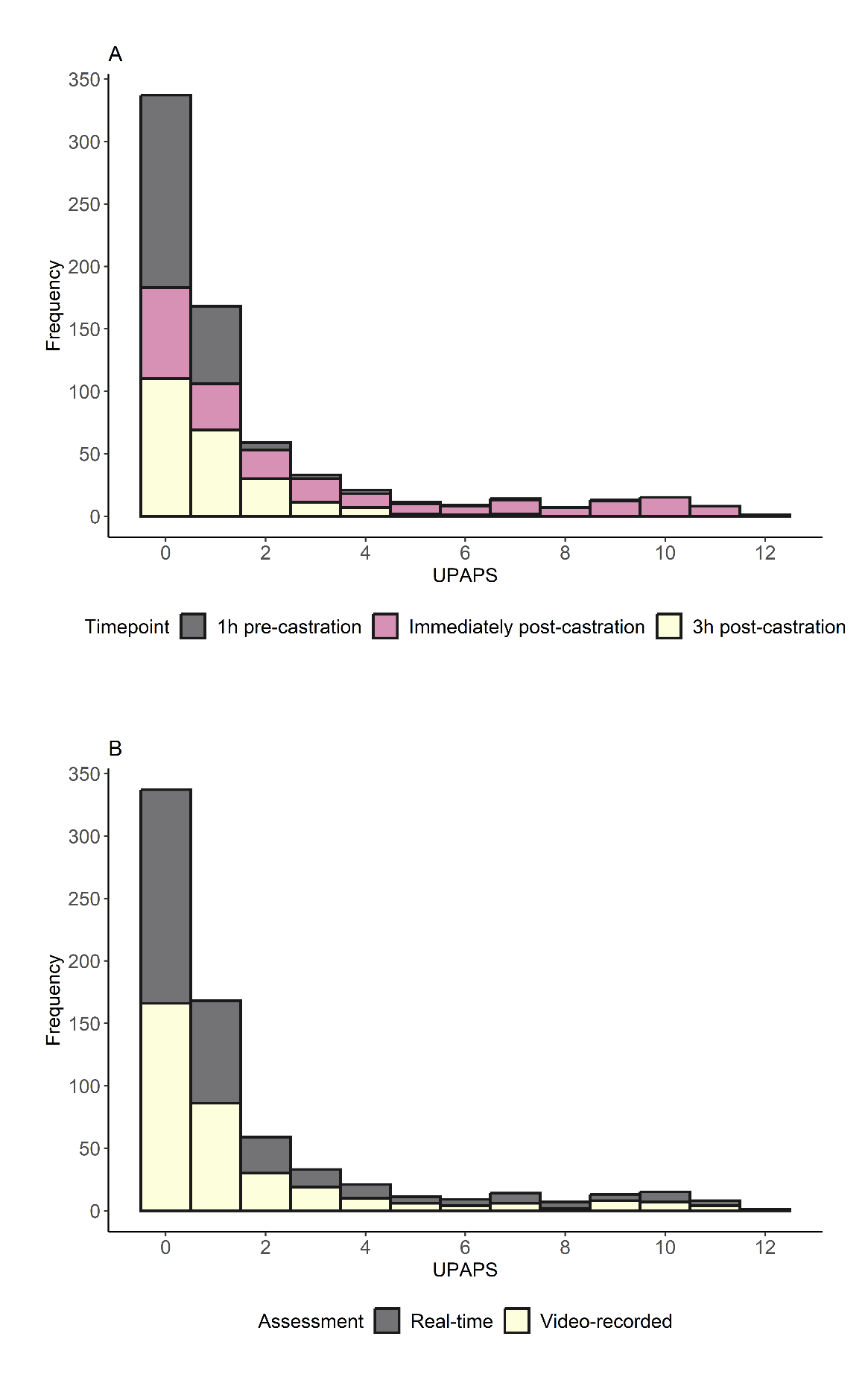


**Figure S1.** Histogram of Unesp-Botucatu Pig Composite Acute Pain Scale (UPAPS) overdispersion per timepoint (A) and assessment method (B).

**Table S1.** Multilevel zero-inflated negative binomial model findings using Unesp-Botucatu Pig Composite Acute Pain Scale (UPAPS) as response variable.

| **Fixed effects** | **Estimate** | **SE** | **Z-value** | **P-value** |  |
| --- | --- | --- | --- | --- | --- |
| Count component of the model | | | | | |
| Linear coefficient (α) | -0.4278 | 0.2819 | -1.5175 | 0.1291 |  |
|  |  |  |  |  |  |
| Slope coefficients (β) |  |  |  |  |  |
|  |  |  |  |  |  |
| 3h post-castration | 0.2375 | 0.2034 | 1.1677 | 0.2429 |  |
| Post-castration | 1.5978 | 0.1958 | 8.1617 | 3.30^-16^ |  |
| AssessmentMethodology | -0.0714 | 0.0919 | -0.7770 | 0.4371 |  |
|  |  |  |  |  |  |
| Logistic component of the model | | | | | |
| Linear coefficient (α) | -1.0111 | 0.5462 | -1.8512 | 0.0641 |  |
|  |  |  |  |  |  |
| Slope coefficients (β) |  |  |  |  |  |
| 3h post-castration | -17.7521 | 2829.3611 | -0.0063 | 0.9950 |  |
| Post-castration | -0.7205 | 0.5946 | -1.2117 | 0.2256 |  |
|  |  |  |  |  |  |
| Random effects | Estimate | SD | Number |  |  |
| Piglet:Litter | 0.1953 | 0.4420 | 29 |  |  |
| Litter | 0.1946 | 0.4412 | 15 |  |  |
| Observer | 0.0869 | 0.2949 | 4 |  |  |

SE is standard-error; SD is standard-deviation

**Table S2.** Simple linear model findings using the differences of Unesp-Botucatu Pig Composite Acute Pain Scale (UPAPS) total score assessed in real-time and video-recorded as response variable.

| **Fixed effects** | **Estimate** | **SE** | **Z-value** | **P-value** |
| --- | --- | --- | --- | --- |
| Linear coefficient (α) | -0.0370 | 0.1397 | -0.265 | 0.7910 |
|  |  |  |  |  |
| Slope coefficients (β) |  |  |  |  |
| Average between assessed methods | 0.0544 | 0.0491 | 1.107 | 0.2690 |

SE is standard-error
